# Extra Supplement.—The Nursing Mirror

**Published:** 1894-02-24

**Authors:** 


"The Hospital" Nursing Mirror.

Being the Extra Nursing Supplement of "The Hospital" Newspaper.

[Contributions for this Supplement should be addressed to the Editor, The Hospital, 428, Strand, London, W.C., and should have the word "Nursing" plainly written in left-hand top corner of the envelope.]

### News from the Nursing World.

### A ROYAL PATIENT.

Princess Ena, the little daughter of Princess Beatrice, continues to make excellent progress, and will soon, it is hoped, entirely recover from the effects of her accident. The fact that the Queen has left Osborne for Windsor is a satisfactory proof that she is no longer uneasy about the little grandchild, on whose account the journey was postponed last week.

### THE HILDA MAY COT.

The Duke of Teck presided at the annual meeting of the Richmond Royal Hospital, the Duchess of York and the Duchess of Teck being present on the occasion. A subscription of £20 from the Queen was announced with many other sums given for the endowment of the Hilda May Cot.

### ANOTHER NEW CLUB.

A most attractive establishment has recently been opened in Tottenham Court Road under the title of "Ideal Club and Restaurant," where excellent food daintily served is provided at wonderfully moderate prices. The pretty ferns on the fresh white tablecloths and other pleasant details are as noteworthy as the charming music which is also provided. The club is open to men and women, and is designed to supply them with recreation, social enjoyment, and intellectual advancement. All particulars can be obtained from the secretary.

### NURSING AT MORTLAKE.

It is very pleasant to hear of the steady progress which has been made by the Mortlake Nursing Institution, affiliated with the Queen's Jubilee Institute. Fifty new subscribers have been added to the list, and Miss Peter after her customary inspection expressed entire approval of the work of the nurses. The financial position of the Mortlake Institution is particularly good, and the nursing has every prospect of continued success in the capable hands of Miss Cross and Mrs. Foster.

### SURE AND SIMPLE.

"I can't imagine how we got all this scarlet fever into the village," remarked the squire, who justly prided himself upon the excellent sanitary arrangements in his labourers' cottages. "It's been puzzling me," rejoined the busy local doctor, "but I picked up the clue yesterday. Your under-gardener's wife had one of her children down with a rash awhile back, and would not let me know. She said doctors were always such 'a worrit'; then the child desquamated, and 'the peeling' was rather unusually marked, so the woman collected the biggest bits of cuticle and took them round to consult other wiseacres as to whether her child could be a leper! After she had visited half-a-dozen cottages, she met a young man who had been laid up in a fever hospital a year ago, and he had the sense to tell me. I have spent the morning investigating, and have tracked the fever home to her in five cases certainly. Your gardener's wife will be confirmed in her opinion that doctors is rare worrits!" concluded the harassed general practitioner.

### HOSPITAL PROGRESS.

The Kent and Canterbury Institute for Trained Nurses has made much progress in the last year. The number of nurses has been considerably increased, and the Association has become completely self-supporting, thanks to the excellent management of the Lady Superintendent (Miss Shaw). Nurses joining the Royal National Pension Fund are assisted in the payment of their premiums by a special fund set apart for the purpose. Full training is now insisted upon for the nurses, and the Kent and Canterbury Nursing Institute may be congratulated in every respect upon its present condition.

### A PLEASANT ENTERTAINMENT.

The nurses at Fir Vale Union Infirmary gave an excellent waxwork exhibition in their large dining hall on February 12th, and certainly the general management of the entertainment was most succesfully conducted. Loud encores made the spacious hall ring again, and the different characters were admirably represented. Miss M. Thomlinson, the lady superintendent, evidently endeavours to brighten the lives of her nurses in all reasonable ways. At the end of the waxwork exhibition various songs were well rendered by the nurses. Mr. B. Young (chairman) and the Rev. Beeby (vice-chairman), with other members of the hospital committee, were present during the evening, and the Governor (Mr. E. Waters) thanked the nurses at the end of the entertainment for the way in which they had catered for the amusement of the audience.

### AM I MY BROTHER'S KEEPER?

Two questions discussed with some spirit at a recent meeting of the Sanitary Authority at Wellington, Salop, had reference to the question of trained nurses at Wrockwardine Wood Hospital, and if nurses were necessary, who was to pay for them? The last question would seem to have been fully answered by Dr. Cæsar, who remarked that only the previous night a woman had died from lack of care, but this fact failed to convince the chairman. He denied that "the profession" had more knowledge than the laity, and considered that the public "should be left to fight as much as possible against the battle of life themselves." Apparently the sick members of the public then under discussion had not made a very successful fight so far. But whatever may be demanded of individuals in health, it is hardly fair to expect them to do battle from their beds; most likely they will have to submit to the unlucky fate which leaves them to die from want of proper care, although they are happy in possessing at Wrockwardine Wood Hospital a doctor who understands and sympathises. Perhaps he may eventually persuade the authorities that some one is morally responsible for all lives sacrificed to the lack of competent nursing.

### A NURSE'S FIRST DUTY.

However much the rules laid down for nurses may vary in different places, it is universally conceded that obedience is the first duty. Until that lesson is learnt a probationer is absolutely useless amongst sick people. It is perhaps difficult to say where disregard of discipline may have the most disastrous consequences, but certainly it should never be overlooked in a person nursing infectious cases. The statements of the Chairman of the Local Government Board at Normanton are somewhat startling, for he asserts that the nurse at the Isolation Hospital mixes carelessly with people whilst she is attending small-pox patients. "She has been warned several times." "She appears to do as she likes, irrespective of all caution," are amongst the remarks made on this subject. Either those officially responsible for public safety are misinformed, or they are themselves neglecting due precautions. If a nurse has so little knowledge of her duty the sooner she is replaced by some one less ignorant the better for patients and their friends. The facts may have been inaccurately reported, and if so the public ought quickly to be disabused of its present impression that Normanton is most inadequately protected from the chances of another small-pox epidemic.

### FRIENDLY SAILORS.

The Grimsby and District Nursing Institution has a good friend in the captain of the large four-masted ship "Falls of Afton." He showed his interest in the welfare of the association on a recent Sunday by allowing his vessel to be opened to the inspection of visitors, donating the small admission fees charged on the occasion to the Nursing Fund. Sailors are deservedly popular patients in hospital wards, and it is pleasant to find the good-will thus substantially reciprocated.

### ON DUTY NIGHT AND DAY.

There are two nurses at Cheltenham Workhouse Infirmary, one of whom has charge of an average of fifteen male patients and the other of the same number of women. Among the latter there are maternity cases, and the nurse is not relieved of her responsibilities during the night; either she must be constantly roused or the patients must be neglected. There have lately been several typhoid cases amongst the male patients, and they also have been solely dependent on the care of a single nurse. The board acknowledge that their two nurses have been considerably overworked, and eight guardians at a recent meeting voted for a qualified nurse for night duty. However, twelve guardians opposed this humane suggestion, and urged that it would be "throwing money away." So the matter was dismissed, and the doctor is to have a telephone to the female wards instead. Whether this machine will prove as practically valuable to recently confined women or to men with typhoid, as the night nursing of a skilled woman might be, is at any rate open to question.

### QUEEN'S NURSES IN WALES.

The last annual meeting of the Bangor Trained Nurses' Institute was held on the 13th inst., when a report of the year's work was laid before the subscribers. A Queen's Welsh nurse has been established at Carnarvon in addition to the two in Bangor; one in Bethesda, one in Menai Bridge, and one at Beaumaris. Miss Vickers has been appointed superintendent in place of Miss Hurst. The subscriptions are still inadequate to meet the expenditure, although they have somewhat increased during the last twelve months.

### ABERDEEN.

The alterations at Aberdeen Infirmary now approach completion, and the results in the increased and improved accommodation for the nursing staff are eminently satisfactory. Electric light has been secured, and bath-rooms, larders, and kitchens are on the most modern plans, and the pretty tiled walls are as pretty as they are sanitary.

### PARISIAN PRECAUTIONS.

Gratuitous revaccination is promptly offered in Paris to the neighbours of any patient suspected of having small-pox, and no pains are spared to impress upon them the importance of the step. The disinfection of rooms in which death has taken place, not necessarily from a nominally infectious disease, is also an excellent plan in which our French neighbours set a good example.

### COMFORTABLE COMPLACENCY.

"I am very glad to see you here," remarked the courteous Rector, making way for the local Mrs. Gamp at the schoolroom door; "I expect the London lecturer will be able to teach us all something!" "Well, sir," responded Mrs. Gamp rather crossly, "O' course, there's no telling. She might know a little something as I don't, so I've just stepped in to see! But I ain't sure, after all, as I don't know a'ready everything as is to be knowed."

### SHORT ITEMS.

At a largely-attended meeting at Chertsey it was unanimously decided to secure the services of a trained nurse for the parish.—The Lincoln Board of Guardians have expressed complete satisfaction with the nurses supplied to their infirmary by the Northern Workhouse Nursing Association.—At a meeting of the sanitary authorities of the port of Barnstaple the excellent work of Nurse Taylor on the hospital isolation ship was warmly commended.—At the annual meeting of the Birmingham District Nursing Society a very satisfactory report was laid before the subscribers.—The Southampton branch of the Queen's Jubilee Institute has held its first annual meeting, and shown a good record of work, but funds are urgently needed to increase the staff of nurses.—A scheme for district nursing amongst the sick poor at Blackburn is under discussion.—The Belfast Maternity Hospital has held its centenary meeting, and presented an excellent record of work. The necessity of additional accommodation was pointed out, and it was arranged that an architect should be consulted before any appeal for funds be made.—The Hampshire Nurses' Institute has received a bequest of £50 from the late Miss Ogden.—The Duchess of Teck has become a patroness of the Cheltenham Home for Sick Children.—It is proposed to start a building fund, which it is hoped may, in the course of five years, justify a reasonable hope of securing new and commodious quarters, and also a garden, which is sorely needed for the children who remain under treatment for long periods, owing to the chronic nature of their illnesses.—It was proposed at a meeting of the Kidderminster Guardians that books should be hired for the use of the sick paupers; the suggestion was negatived, but, probably, residents in the neighbourhood now realise the enormous boon that a constant supply of books and papers would be to inmates whose days are so terribly monotonous.—The Hull District Nursing Association held a successful annual meeting in the Town Hall.

### On Nursing the Nervous and Insane.

By T. DUNCAN GREENLEES, M.B.Edin., Medical Superintendent, Grahams Town Asylum, South Africa.

### VII.—DEMENTIA OR MENTAL ENFEEBLEMENT.

The tendency of all cases of acute insanity is towards dementia, and it is the object of all treatment to prevent this. Many cases of mania and melancholia, especially when there is a hereditary taint, when they do not recover, pass into dementia. In such cases the brain becomes exhausted, or "played out," from prolonged and abnormal excessive energy; it is unable to recover its former powers, and then becomes enfeebled and the mental faculties become permanently deadened. Acute dementia is usually due to a well-defined disease of the brain; its onset is, as a rule, sudden, and occasionally in these cases recovery takes place. Chronic dementia is that form of disease so well known in asylums, as it forms perhaps the larger proportion of the entire population of any ordinary asylum. It generally follows an attack of acute insanity when the brain storms have been of such severity as to irreparably injure and destroy the brain functions. The patient after a long bout of excitement, during which the nerve exhaustion is extreme, gradually settles down and becomes quieter. Instead of this quietening down being accompanied by an awakening of the intellectual faculties, as would occur in recovery, the patient has a stupid, dazed expression, and his conduct is depraved. He will not speak, he seems deprived of feeling and intelligence; his movements become automatic, and he is generally found standing about with his head hanging down, staring vacantly, and perhaps with the saliva dribbling away from his mouth. When questioned he will not answer; his memory seems destroyed; he cares for nothing; his best feelings and emotions are dulled or obliterated altogether. Told of his wife's death, whom he formerly loved dearly, no notice is taken; his features remain immovably stolid. If the patient is a woman, and her child is brought to her, she may look at it, but no smile of recognition lights up the set countenance. The maternal instinct — that most powerful of all the emotions—is absent, and nothing seems to arouse in these patients a feelingof interest, grief, or pleasure. The mind, in fact, is a complete blank, and external influences fail to make any impression upon it. The eye can see, the ear hear, but the brain fails to appreciate the beauty of the picture or the melody of the music! They are frequently depraved in habits, and negligent and untidy in dress. While the disease may be hopeless when the prospect of recovery is considered, much may be done by intelligent nursing and sympathetic companionship to improve the habits and disposition of such patients. Dotage is dementia, resulting from old age, and is a natural process. The mind, like the body, lives a certain time, and then the process of dissolution slowly supervenes, the mental faculties returning to childhood. The nursing of such cases is an excellent test of the resources and patience of the nurse. Good temper, patience, firmness, and tact are all necessary in a marked degree, for there is no disease so difficult to nurse as senile dementia.

(4) *General Paralysis of the Insane.*—This form of mental disease is peculiar as it embraces in its various stages or types the different forms of insanity already considered. The question is still an open one whether the organic disease of the brain found in such cases is the cause of the mental disturbance or its consequence, or whether the cause of the disease is to be searched for in some outside influence, moral or physical. The patient is excitable as a rule at the outset of the disease, impatient of control, and possessing the most exaggerated ideas of his own wealth, powers, and abilities. After a time of excitement, lasting generally from a few weeks to a few months, he passes into a stage of quiescence or quietude, during which he becomes stout, stolid in expression, and his intellectual faculties are dulled or blunted. When questioned closely during this stage he still confesses to grandiose delusions. Later on he becomes demented—mind and body become complete wrecks; he is unsteady in his gait, staggers about like a drunken man, is very quarrelsome and irritable. He rapidly gets thinner and more feeble, then bedridden, and, unless carefully nursed, bedsores form. He is at this stage in a hopeless and helpless condition, yet he is happy; so shaky are his hands that he is unable to hold a cup to his lips. He is yet the strongest man in the world; while in rags and extremely dirty every piece of paper represents to his distorted imagination a bank note, and every piece of metal he picks up, such as buttons, &c., are sovereigns to him. Later on epileptiform seizures take place, and death draws a curtain over the unhappy scene. The disease is necessarily fatal, no remedy having been yet found for it, and its duration is for from two to three years, and rarely this period is prolonged, especially in women, for several years longer.

It is essentially a disease of adult life, and attacks its victims at a time when the brain should be in the full zenith of its power. While most frequently due to dissipated habits, it may be caused by mental worry, such as business anxieties, and it attacks men much more readily than women. Although the duration of the disease is really very short as a rule, still by good nursing, careful feeding, and a constant attention to cleanliness, the fatal termination may be delayed for some considerable time.

(5) *Insanity Associated with Epilepsy.*—Epilepsy is a disease to which many persons are subject who are yet not legally insane, but if the disease is of long duration the mind gradually gives way, and outbursts of maniacal disturbance takes place. It is a fine legal question to decide where epileptic sanity ends and where epileptic insanity begins. The decision is generally made for us when the patient commits some outrageous act. It is well nigh impossible for any person subject to severe epileptic fits to possess a well-balanced mind. The terrible explosions that take place within the cutical cells producing an ordinary fit, of necessity disarrange their usual functions, and the confusion of mind succeeding the seizure is undoubtedly due to temporary mental instability.

When well marked symptoms of insanity are found associated with epilepsy these symptoms take on definite types. Then we may have epileptic mania, epileptic melancholia, epileptic dementia, when the mental faculties have been permanently injured and destroyed by the epilepsy, and epileptic imbecility, when during childhood the growth of the mental faculties has been arrested by epilepsy.

The symptoms of a fit are familiar, but the mental characteristics of epileptic insanity depend upon the form of insanity seen in any given case, and the natural tendency of ordinary idiopathic epilepsy, where a cure is impossible, like other forms of acute mental disease, is towards dementia.

To a nurse whose ambition it is to undertake the care of the insane such a subject as their habits should receive attention. This, however, is well done in the second edition of "Handbook for Attendants on the Insane," published under the supervision of the Medico-Psychological Association, and the duties of a nurse who undertakes such work I endeavoured to describe in a lecture published in THE HOSPITAL of May and June, 1889.

In conclusion, I would impress upon all that nursing and caring for the insane is no mere child's play. Nurses must bring to the work all their energies, capabilities, patience, and tact.

It is a trying life, worrying in the extreme, and not entirely free from danger. The reward looked to is not praise of men, but the pleasure of seeing of recovering patients the gradual dawn of a new mental day, the awakening to life of a mind which, perhaps for months, has been clouded over and, to all intents and purposes, dead.

Such an experience is a frequent one, and surely such studies are worthy the attention of the philosopher; at any rate, they are reward for which medical superintendents, as well as nurses, are content to work.

### District Nursing.

### VIII.—MATERNITY WORK (*continued*).

### Care of the Infant.

If a dependable monthly nurse or skilled friend is attending, the washing of the baby may be left to her, but if there is any doubt the midwife will be wise to wash it herself until the cord has separated. In any case she must attend to the cord daily, and to the eyes, which need careful bathing with plenty of warm water. The first sign of inflammation must be shown to the doctor for special treatment. It is imperative upon a midwife to seek medical advice without delay if any unfavourable symptoms show themselves in either mother or child, and also, of course, should any complications occur during labour, or before and after.

### Duties of Monthly Nurses.

The chief duties of a monthly nurse are strict obedience to the doctor's orders and ensuring perfect cleanliness of the patient. No lying-in case should be touched until the nurse has washed her hands thoroughly with soap, water, and nail-brush, and then disinfected them in 1·2000 perchloride of mercury or creoline and water (1 dr. to 1 pt.). Soiled linen must never remain in the room. It should be at once removed, and placed in cold water.

### The Daily Report.

A full report of every point concerning mother and child should be written daily for the doctor, and temperature and pulse recorded twice daily for at least six days.

### Washing and Dressing the Baby.

There is a decided art in washing and dressing an infant quickly and well. Every maternity training school has its own rules, but all agree that the child should be turned as little as possible, and that the time should be as short as is compatible with thoroughness in details. A good maternity nurse gets everything ready before she takes the baby. The bath (often in an absurdly small basin, though the best procurable) is made the right temperature (90°), the clean garments put ready before the fire, the towel, soap, and flannel conveniently near, also boracic lotion for the eyes (if necessary), the squares of linen for eyes, nose, mouth, and cord, the powder and threaded needles, thimble, scissors (round pointed), and safety pins. Finally the nurse puts on her flannel apron and commences operations, taking care to sit so that the child's feet (not its head) are towards the fire. If clean binders are needed they should be rolled beforehand; if not, those on the child should be well aired before being put on again. The friends must be warned never to use soda in washing the infant's napkins, as this causes chafing and irritation of its delicate skin. (They must also be thoroughly well rinsed after washing or the child is sure to suffer.)

In dressing the baby, the nurse should so put on the calico binder that the body of the long flannel garment remains well up to the neck both in front and at the back, for too often the chest and back are left uncovered, except for the outside gown.

### Diet of Mother and Infant.

The mother's diet will be directed by the doctor, but if a midwife is in sole charge she must arrange that it is easily digestible for the first two or three days; after that time ordinary food may be resumed. A simple aperient is required not later than the third day, and by then lactation is generally established. Care must be taken that the unfortunate infant is not fed with sugar and butter, gruel, cow's milk, &c., by its so-called friends. It usually needs nothing beyond being put to the breast twice or thrice daily for the first two or three days. It then should be fed regularly every two or three hours, the intervals being rather longer at night. If absolutely necessary, a little cow's milk with three parts of warm water, slightly sweetened, may be given. Keeping the child constantly at the breast is a fruitful source of sore nipples, which too often lead to mammary abscess. When a midwife or nurse has the opportunity of seeing her patient before the confinement, she should advise that the nipples should be bathed at least twice daily with brandy and water or gin and water, to harden the skin. If soreness arises after confinement, they must be bathed with warm water each time the child is suckled, and the application ordered by the doctor applied. Boracic lotion is a useful application, or a solution of tannic acid, but whatever is used must be washed off before giving the child the breast.

### A Simple Support.

Sometimes the fulness and tenseness which occur at the beginning of lactation can be relieved by supporting the breasts for a time. A simple and efficient support is easily made by a small square of linen with tapes sewn to each corner. Two are tied round the waist, two round the neck, in such a way that the breast is firmly and evenly supported. A fold may be made at the top of the square and secured by a safety pin, so covering the breast completely. This is also useful when frequent poultices or fomentations are ordered, as it is only necessary to untie the strings round the neck to change the application.

Every patient should be warned against the use of stimulants during the lying-in period except by special order from the doctor.

### Reasonable Precautions.

In case any suspicion of blood-poisoning should arise, both midwife and nurse must be guided by the views of the medical man as to beginning maternity work again. Most careful cleansing and disinfection of everything that has been exposed to infection is necessary. Mere abstaining from this work for a certain time is of no real value without every other precaution is also taken.

District nurses should discourage free midwifery, except under special circumstances. Parents should be taught to realise their responsibility in this matter, and be encouraged to prepare for their children.

Unless when specially desired, it is better for the doctors to take the cases, and then the nurses can attend under their directions without further expense to the parents, and render invaluable help. In any case, the patient secures a woman to do the washing, look after the children, home, &c., and if the nurse takes entire charge of mother and infant they are saved from neglect or ignorant interference.

### Example and Precept.

The nurse can not only carefully carry out the wishes of the doctor, but she can show the friends the necessity of cleanliness, proper feeding, &c., and give valuable object lessons on the points which every woman ought to know who wishes to help her friends at such times. The main thing is to make them understand there are reasons for such ways, that they are not mere fads of nurse or doctor. The right way is just as easy as the wrong in most things, and a few practical demonstrations with regard to beef-tea free from fat, thin, smooth gruel, for instance, to say nothing of more important details of cleanliness, &c., for mother and infant, will make not only that patient more comfortable, but affect others, and lead by degrees to a better knowledge of these simple facts. A nurse must keep on, "line upon line, precept upon precept," in this work, remembering she is fighting against generations of ignorance and tradition in this special branch of her profession, and so not being easily discouraged by the frequent failure of her efforts to introduce better things.

### Lectures at St. George's Hospital.

### THE HISTORY OF THE HOSPITAL.

THE first of a course of fourteen lectures to nurses was given at St. George's Hospital by Mr. C. T. Dent in the board-room of the hospital on Friday, February 16th. The subject of this and the following lecture was "The History of the Hospital;" the story of its foundation and progress.

St. George's Hospital was the happy outcome of a quarrel. An older hospital, the Westminster Infirmary, situated in a street called Petty France (famous as the home of Milton), had to be enlarged, and two sites were proposed, one near the original building in Pimlico, the other in a "remote spot in the country"—Hyde Park Corner. The governors were much perplexed between their rival merits. In the words of a quaint minute of the day, there were "heats and differences" on the subject, but the affair ended in the "country" site—Lanesborough House—being started as "St. George's Hospital," while later the Westminster Infirmary was moved to the site in Pimlico. Lanesborough House was opened, with thirty beds, in January, 1734. There were few houses adjoining, none on the south side, and, indeed, one of the chief objections urged against the site in the first instance was its lonely position, far from "town," *i.e.*, Westminster, reached by a dangerous road infested with footpads.

Lanesborough House, however, was a success, and subscriptions came in rapidly. In 1735 the beds were reduced to twenty-five, but a few years later the authorities found finances in so satisfactory a condition that it was possible to support 100 more patients than could be accommodated. It was then decided to purchase the freehold of Lanesborough House for the sum of £500. It is not certain how many beds the hospital actually contained at this period, but in 1744 about 100 beds were in use, and in 1770 they had increased to 200. A little later the beds were reduced to 125. At this time the inscription over the entrance read: "St. George's Hospital for Ye Sicke and Lame. Supported by ye voluntary contributions of ye nobility, gentry, and others.' "It seems to me," said Mr. Dent, "that now the inscription should run: 'St. George's Hospital, which ought to be, but is not, supported, &c.'"

In the original hospital the nursing staff numbered ten, that is one nurse to every three beds, a proportion very much the same as now. Various notes of nurses taken on at this time show that the average rate of wages was £6 a year, plus gratuities, varying in amount from 5s. to £4. When nurses did not give satisfaction the weekly board were authorised to settle the gratuities. The matron's salary was £10 a year. It is tolerably clear that discipline in those times was a little lax, nursing questions constantly occupying the attention of the weekly Board, the governors realising the importance of such matters. In 1739 a rule was made that notice was to be given to the porter not to suffer any nurse to go out, "on any pretence whatsoever," without leaving a ticket with him. Judging from information in the old minute books, Mr. Dent thought the modern idea that nurses in those early days were unskilled was unfounded, various entries occurring relating to the dismissal of nurses as being unfit for the discharge of their duties. The Apothecary of those days was a most important person. Here, as elsewhere, he was in charge of the bleeding and cupping. Few patients passed through the hospital in those days without submitting to one or other of those operations. If a patient's case was not clearly made out, as not infrequently happened, he was bled on general grounds. The last man to hold the appointment of Apothecary was Mr. John Hammerton, who resigned in 1866.

Mr. Dent was afraid nurses would be surprised and possibly shocked to hear that their predecessors differed so much from present day nurses that they actually had grievances, chiefly on the subjects of beer and wages. In consequence of their representations the wages were raised in 1748. With regard to beer, the potman from the nearest public-house made his rounds as regularly as the house physician, touting for orders, but in 1782 it was ordered that no beer or strong drink be allowed to be brought in for the patients except by permission of the doctor. In 1789 the Board found it necessary to order the lower sashes of the ground floor windows to be so secured as to make it impossible for food and drink to be passed in from the outside to the danger of the patients.

### THE NURSING DEPARTMENT.

In 1747 the nursing staff numbered about 18 and the beds 100. In 1749 the nurses were increased to 20. In times of emergency, and on special occasions, as for cases of small-pox, extra nurses were engaged, who were probably quite isolated with their patients and provided their own board. Entries in the minutes are found naming the sums paid to nurses for special cases. Nurse Rummit is mentioned many times in the minutes. She was a nurse of the "Salivating Ward," and evidently an important personage. In those days every hospital had its salivating ward for patients who were undergoing a special form of treatment, almost as popular then as bleeding and cupping, namely, the administration of large doses of mercury, resulting in great weakness and other unpleasantnesses.

It is usual nowadays to assume that nurses in the last century were drawn from a very low class of women, but though this may have been the case with the "inferior nurses and servants," the nurses in charge of the wards appear from the minutes to have been chosen with some degree of care and to have been very capable women on the whole. In these days nurses are popularly supposed to be either Gamps or angels, but Mr. Dent confidently assured his audience he was equally far from holding either belief. In 1743 a careful list of the nurses employed by the hospital was kept and duly signed by the board and the chairman, and special mention is made that "no undue preference" shall be shown in filling any vacancy. Fifty years after the hospital was opened an advertisement was put in the *Daily Advertiser* for women of "good health, between 30 and 40 years age, able to read writing," such to apply to Mrs. Horn, matron. No "training" of any kind being considered necessary. But the matron had little to do with the management of the nursing, clearly far less than had the apothecary. Her duties were multifarious, but she had nothing to do with the engagement of the nurses, and no power to suspend or discharge them, though responsible for the "inferior servants." In 1733 the rules for the matron included "the care of the keys of all the doors," and she was required to see that the locking of the front door was attended to at particular hours, varying according to the season of the year. Further, she is on no account to allow patients to go into the Green Park or St. James's Park, these places being fashionable resorts, it being probably feared they might cause annoyance by begging. Any misdemeanour on the part of nurses or patients the matron is expected to make known to the board forthwith, the Board at that time evidently concerning itself with very small details indeed, such as the food provided for the "family dinner," the matron having the power to vary the dinner on Sundays. When salt meat is given she was permitted also to provide fresh meat "for such as do not like the salt." The matron is also enjoined to see that the "apothecary hath a bit of fresh butter every day to breakfast if he desires." By a special order of the weekly board in 1740 the matron is empowered to provide "a paile" for a certain nurse. There is a note in the year 1756, one Mrs. Abigail Hampstead being matron, that the house surgeon or apothecary's pupil, the matron, and all the senior nurses, are called before the weekly Board, and mutual "decency and civility" enjoined between them. On the first occasion when this was done, the famous John Hunter was house surgeon. It 1825 the rebuilding of the Hospital was determined on, and its history from this point will be dealt with in the next lecture.

### Old-Time Nurses.

### II.—A BRAVE LIFE.

The course is nearly ended now, and the brave life is drawing towards the shadows of evening. It is freed from the pressure of care by the thoughtful kindness of those who have made provision for the labourers worn out in this special field of work, and little remains but to linger out a peaceful age in thankful expectation of the great change. She who has soothed many a dying hour and witnessed many a scene of patience and glad faith in extreme suffering can have no dread of the last great mystery. Death to her is a familiar presence—not always or altogether an unwelcome one.

The brave life is in many respects a typical one of this class of "old-time" nurses whom we are in danger of thrusting on one side in the battle of life, and its incidents are not without instruction.

Nurse W. was of no ordinary parentage. Her father, an Austrian Pole, had taken part in the Battle of Waterloo under Blucher, and speaking seven languages, had subsequently served as a courier, in which capacity he fell in love with and married an English girl. He died shortly before his third child was born, and is but a dim memory to his eldest daughter, who still keeps on her wall the spurs which he wore in the great battle. The mother was a deeply religious woman, of small education, but "from Genesis to Revelations she could tell the contents of any chapter in the Bible." The children early had to work for themselves, and until the age of thirty Nurse W. remained in service as a lady's maid. Chance had brought her in touch with much illness, and an increasing love for the work of nursing induced her at that age to enter Queen Charlotte's Hospital and qualify for a midwife or monthly nurse. The instruction given was fragmentary enough. She learned what she liked by observation, and picked up sufficient knowledge to procure a diploma as a duly qualified nurse. Those were the days before the present building had been erected, and when as many as ten or twelve beds were accommodated in each ward. Monthly nursing was not then, as now, a profession apart, and Nurse W. from the first took all cases, surgical or medical, which came in her way. It is characteristic of her interest in her profession that she obtained a certain amount of knowledge about the dressing of wounds, &c., from visits to King's College Hospital by the contrivance of a friend, who was a nurse there. In this manner she studied from time to time, whenever she could gain admission to the wards, the more important details of surgical nursing, and having quick wits and an excellent memory began to be known as a skilled and safe attendant. It was then that a break occurred in her nursing career which threatened to deprive her of any chance of success. During a trying illness of her mother's, extending over several years, Nurse W. gave up every other engagement and undertook the care of her with entire devotion until the end. Such a long-continued break as this lost her, of course, the connection which she had slowly and laboriously built up, and, moreover, consumed any savings she had been able to make up to that time. It is a special feature of the nursing life that it so often presents these highest sacrifices to those who follow it. In any sickness or sorrow the nurse member of the family finds her services claimed with loving insistance, and will commonly render them with an unworldly disregard of self-advantage which has become typical of her calling. Others may be as willing, but it is she who is able, fitted by the daily discipline of self-effacement, to be the mainstay of those in trouble, and many a staid, elderly woman finds true gladness in lonely hours from the memory of some honourable post relinquished or favourite work transferred to other hands at the call of kindred or friendship. These are the living sacrifices which make life worth living.

To return to the nurse. It was uphill work beginning all over again, and only by slow degrees did engagements become at all plentiful. There were many long intervals of months when she waited, eating her heart out in suspense, for the summons which never came. She lodged at this time and always afterwards with her sister, who was struggling unsuccessfully under the burden of a small shop, and it is evident that as work increased by far the larger share of the mutual income was contributed by the nurse. Chance brought her at last under the notice of a physician of wide repute, a relative of whom she had nursed back to life under circumstances of unusual difficulty, and this brought about at length the desired introduction. She was told that her future lay in her own hands, and asked which branch of her profession she preferred, to which, with old fashioned reserve and submission to superiors, she replied that having her living to gain it did not become her to choose. The result of it all was a recommendation to a surgeon who has since gained one of the highest reputations of the day, and under him she was speedily occupied without intermission. Advocates of an eight hours day for nurses would open their eyes at the story of the work expected from the "old-time nurse." After the most critical operation one nurse only was placed in charge of the patient, who was expected night and day, with no definite hours for sleep, much less for recreation, to do all that was required. It was no unusual thing for her to be three weeks without taking off her clothes, getting restless snatches of sleep in a chair by the patient's bedside under the constant sense of responsibility, and to be then despatched to an equally urgent case without a day's interval, while a less experienced nurse was placed in charge of the convalescent. It needed an iron constitution to support this life year by year. But this the "old-time nurses" seem to have had. Above all they were sustained by intense love for their work, and a healthy pride in their own skill and that of their special surgeon. Many operations which are now a matter of course partook then more or less of the nature of experiment. The nurse could often directly supplement the experience and observation of the surgeon, and was conscious that her judgment and accuracy were important factors in the deductions to be made.

The payments of the old-time nurses were not excessive. For some years a guinea a week was all that Nurse W. received for the most critical day and night cases. Later on she received from 30s. to £2 2s. a week, and once or twice touched the high water-mark of £10 for a month. People were accustomed to say they wished they were rich enough to reward her services at their true worth. That was the stock formula, and probably it has not much changed since. But the nurse may be excused for feeling that "this remark, however gratifying to the feelings, does not fill the pocket." There was no fixed scale of fees. Like Sir Andrew Clark, she took what was offered, and made no complaint, and the result, liberally shared as it was with her sister, came very far short of securing her old age against want. In the course of her long experience (for she continued nursing up to the age of seventy) all sorts and conditions of men passed under her observation. From palaces to hovels she marked every variety of life with quick eye, reticent tongue, and unfailing memory, and now, as she looks back on it all, missing the cultivated talk, and longing sometimes for a little escape from commonplace surroundings, she sums up her experiences in the words of the melancholy Jacques, "All the world's a stage, and all the men and women merely players."

### Wants and Workers.

A District Nurse will be very glad of old linen or worn underclothing for a poor woman suffering from cancer. Mrs. M., 82, Victoria Street, Ashton-under-Lyne.

### News from Toronto.

BY OUR TORONTO CORRESPONDENT.

THE Training School attached to the Toronto General Hospital has recently adopted a new and pretty uniform, and, as it was found impossible to complete this change in time for the usual annual gathering, the distribution of certificates and badges took place privately. The report reads as follows: Applications for admission, 413 formal and 125 personal. Thirty-nine of these candidates were admitted, but only twenty-two proved satisfactory, and after completing the month of probation were accepted as members of the school. The total number of nurses now holding the certificate of this school is 181, twenty-five of these having passed their final examination, and completed a two years' course during the first year.

Lectures and classes have been conducted semi-weekly from October until July, these lectures being given by many prominent physicians and surgeons, while the classes were held under the direction of the Lady Superintendent.

Examinations are conducted every six months, and each nurse is obliged to obtain at least 50 per cent. in each subject in order to pass.

It is gratifying to record that a large number of the nurses trained in this school are now holding hospital positions both in the United States and Canada, while many others are engaged in private nursing, and some have gone to India, China, and Africa.

The following is a list of those who received appointments during the past year: Miss M. Tye, Lady Superintendent General Hospital, London, Ont.; Miss R. R. Pish, Lady Superintendent General Hospital, Collingwood, Ont.; Miss E. McKenzie, Lady Superintendent General Hospital, Owen Sound, Ont.; Miss L. Sheppard, Lady Superintendent General Hospital, Guelph, Ont.; Miss A. Boyd, Lady Superintendent General Hospital, Morden, Mon.; Miss K. Underhill, Lady Superintendent Children's Hospital, Toronto; Miss H. Hollingworth, Superintendent Nurses' Seeney Hospital, Brooklyn, N.B.; Miss S. Synden, Superintendent Nurses' City Hospital, Albany, N.B.; Mrs. A. Bolton, Matron Lumberman's City Hospital, Brainend, Minn.; Miss A. Haight, Assistant Superintendent General Hospital, Toronto; Miss J. Turner, Assistant Superintendent City Hospital, Erie, Pa.; Miss A. McRea, Supervisor Night Nurses General Hospital, Toronto; Miss A. J. Scott, Head Nurse Pavilion General Hospital, Toronto; Miss A. Newman, Head Nurse Alexandria Hospital, Vancouver, B.C.; Miss E. Dawson, Head Nurse Railroad Hospital, Brainend, Minn.; Miss E. Jones, Head Nurse City Hospital, Cincinnati, Ohio; Miss J. Scott, nurse Royal Victoria Hospital, Montreal; Miss A. Hague, nurse Royal Victoria Hospital, Montreal; Miss H. Mellville, missionary, sent by American Board of Foreign Missions to Central Africa. The health of the school has been such as to call for much thankfulness—not one case of serious illness having occurred during the past year. The library has been increased by the addition of forty-seven volumes, presented by the Ancient Order of Foresters. The recreations of the school have been further promoted by the thoughtfulness of the Commercial Travellers' Association, who sent complimentary tickets for their annual concert, and by the ladies who provided the necessary funds to meet the expenses of the social evening at the Home during Christmas week. This occasion was an exceedingly pleasant one, and will long be remembered. The entertainment consisted of recitations, vocal and instrumental music, and refreshments. There was also a little souvenir for each nurse in the shape of work baskets, pin trays, &c.

List of graduates for 1893: Miss Maigh Cruickshank, Niagara Falls; Miss Euphemia McKenzie, Wiaston, Ont.; Miss Grace Hodgson, Toronto, Ont.; Miss Lizzie McLelland, Peterborough, Ont.; Miss Jessie Green, Bracebridge, Ont.; Miss Jennie Stirton, Guelph, Ont.; Miss Julia Stewart, Kirkwell, Ont.; Mrs. Christina Mounsey, Hoodbridge, Ont.; Miss Alice Newman, Owen Sound, Ont.; Miss Adeline Page, Concord, Ont.; Miss Agnes Scott, Aurora, Ont.; Miss Jennie Halliday, Perth, Ont.; Miss Fanny Ferrier, Toronto; Miss Ethel Dawson, Collingwood, Ont.; Miss Annie Anderson, Whittington, Ont.; Miss Annie Dick, Brampton, Ont.; Miss Lizzie McDonald, Peterborough, Ont.; Miss Bessie Dickie, Hespeller, Ont.; Miss Kate Sutherland, Orangeville, Ont.; Miss Augusta Carman, St. Catharine's, Ont.; Miss Mary Jones, Toronto; Miss Jean Scott, Wyebridge, Ont.; Miss Ida Sharp, Ida, Ont.; and Miss Nellie Miller, Adelaide, Ont.

### Trained Nurses at Bangor.

ANNUAL MEETING.

THERE was a very large attendance at the annual meeting of the Bangor Institute of Trained Nurses, and amongst those present were: Lady Penrhyn, Hons. Alice and Violet Douglas Pennant, Mrs. Assheton Smith, Lady Isham, Colonel the Hon. W. G. Sackville West, Miss Sackville West, Sir Llewellyn and Lady Turner, Miss Wynne Jones, and many others.

The Mayor (Alderman Cameron) was in the chair, and said he had been so much impressed by the good work done by the institution that he intended to give it every support in his power. He felt that the work was so hard and monotonous that the public could not do less than give liberal support to those who undertook it.

Miss Hughes, Superintendent of the Metropolitan and National Nursing Association, Bloomsbury, gave an admirable address, which was heard with deep interest. She said that she considered the reports of the district nursing at Bangor and at the Branch Homes particularly encouraging, and on an excellent system. She spoke earnestly of the golden rules which should be observed by those engaged in this work, especially in intercourse with the poor. They should be encouraged to look upon the nurse as a friend to the family. Her suggestions would then receive an attention which they would fail to obtain if issued in the form of commands. Miss Hughes spoke most wisely on the subject of ventilation, proper feeding of children, and obedience to the doctor's orders. Nurses, she said, must specially avoid the temptation to become "amateur prescribers." As regarded the Mayor's assertion that nursing was monotonous, she altogether disagreed with it, for no woman who had her heart in the work could possibly find it so. Dr. E. J. Lloyd, Dr. Grey Edwards, Dr. Langford Jones, and Dr. E. O. Price all spoke most cordially of the work of the Queen's nurses and of its value. Mr. W. A. Drew, ex-Mayor of Bangor, said that he thought some circular should be printed setting prominently before the public the work of the Institute, which ought to be much more widely understood and appreciated. Mr. Drew also suggested that the admirable address which Miss Hughes had kindly come from London to deliver should be printed and well circulated. Sir Llewellyn Turner spoke strongly of the value of district nursing, and Colonel Sackville West proposed a vote of thanks to the Mayor for presiding, with which the proceedings terminated.

### Minor Appointments.

GOVERNMENT HOSPITAL, CAIRO.—Miss Edith Mawe has been appointed Sister to the Egyptian Hospital, Cairo, and takes up her work there at once. She was trained at St. Bartholomew's Hospital for one year, and was afterwards Sister at the Royal Chest Hospital, City Road, and at the Colonial Hospital, Gibraltar. Miss Mawe held the post of Lady Superintendent at the Dunedin Hospital, New Zealand, and has been well known in the nursing world during the last ten years. She carries with her our sincere good wishes and those of a large circle of friends.

### A Lecture on Cholera.

Given by Professor Klein, at the London Institute.

On 15th inst., an exceedingly interesting lecture was given at the London Institute by Professor Klein, who began by describing the methods used for the prevention of the spread of cholera. As an instance of the success attending these preventive measures, he quoted the Pilgrims' Camp at Hurdwar, India, where in 1891, at a time when cholera was rife in other parts, two cases only occurred. The infection in each case was brought from a distance, and by prompt measures of isolation and disinfection, its spread was immediately checked.

He then went on to speak of the European cholera epidemic of 1892-3, singling out England and Germany as the only countries where it had been successfully combated, at the same time remarking that there were still many localities in these countries where the sanitation was so defective that it was only good luck that had withheld from them such a visitation as had occurred at Hamburg, and on a smaller scale, at Grimsby.

With regard to the isolated cases which had occurred in England, Professor Klein said that some authorities had disputed their being true cholera cases, on two grounds: 1. That they were not epidemic in character; 2. That the source of infection could not be traced. To these objections he replied that an epidemic character was not a test of cholera, and that each case must be judged by its symptoms, and by analysis alone. In support of this he gave the clinical history of several sporadic cases, indistinguishable from true cholera, supporting this evidence by slides showing the bacilli obtained from the intestines of these cases. He maintained, also, that even if the source of infection could not be traced, any case which exhibited the symptoms of Asiatic, as distinct from English cholera must be treated and considered as such.

The latter part of the lecture was chiefly devoted to explaining, by means of slides, the various forms taken by Koch's comma bacillus.

### Professor Horsley on Trephining in the Stone Age.

In the lecture which he gave at Toynbee Hall on the 17th inst., Professor Horsley stated that it was an undoubted fact that the operation of trephining was carried out by the savages of the Stone Age, as it is among the natives of Africa at the present day. The condition of many of the skulls on which the operation had been performed showed that the patient had survived for perhaps many years, and which precluded the explanation that the holes had been made after death. Professor Horsley gave it as his opinion that trephining was usually practised among the Stone Age savages to relieve pains in the head arising from a blow or epileptic fits, the idea being to open the head to let the evil spirit out. Among the Peruvians it was probably practised to relieve the neuralgic pains common in a malarial country.

Professor Horsley went on to say that from the fact of the pieces of bone taken from the head having been pierced and worn as amulets, a theory had been advanced that trephining among the ancients partook of the nature of a religious ceremony, performed on such occasions as admission to the priesthood. For his part, however, he was inclined to regard the operation, even in those early times, as a surgical one to which the savages, with their habitual contempt of pain, had no hesitation is submitting; even though it had to be performed with flint saws and drills.

### Appointments.

Jenny Lind Infirmary.—Miss Row has been made Lady Superintendent of the Jenny Lind Infirmary. She was trained at the Children's Hospital, Shadwell, and at St. Bartholomew's Hospital, and has held the post of Sister at Shadwell since 1886. We wish Miss Row every success.

### For Reading to the Sick.

**Motto.**

We can only elevate ourselves towards God through the souls of our fellow-men.—*Mazzini.*

**Verses.**

There is no sorrow, Lord, too light
 To bring in prayer to Thee;
There is no anxious care too slight
 To wake Thy sympathy;
Thou Who hast trod the thorny road,
 Wilt share each small distress;
The love which bore the greater load
 Will not refuse the less.
There is no secret sigh we breathe
 But meets Thine Ear divine,
And every cross grows light beneath
 The shadow, Lord! of Thine.

—*Jane Crewdon.*

Let us no more contend, nor blame
Each other, blamed enough elsewhere!—but strive,
In offices of love, how we may lighten
Each other's burden, in our share of woe!

—*Milton.*

**Reading.**

It is part of the faith that, since nothing is of chance, He, 'without Whom not a sparrow falleth to the ground," appointeth each slightest accident of thy life. He with Whom "the hairs of thy head are all numbered," knoweth every throb of thy brow, each hardly-drawn breath, each shoot of pain, each beating of the fevered pulse, each sinking of the aching heart. Receive then what are trials to thee, not in the main only, but one by one, from His all-loving Hands; thank His love for each, unite each with the sufferings of thy Redeemer, pray that He will thereby hallow them to thee. Thou wilt not know now what He thereby will work in thee, yet, day by day, shalt thou receive the impress of the likeness of the ever-blessed Son, and in thee, too, while thou knowest it not, God shall "be glorified," yea, and "shall glorify thee."—*Dr. Pusey.*

The sympathy of the Son of Man is many times a hidden sympathy. Whenever, as regards ourselves, it is so veiled that the eye of faith can hardly discern it, then we must think upon the outward expressions of it given, while He was here, as pledges to us of its unfailing continuance. Those human tears, shed on the Mount of Olives and at the grave in Bethany, declare the heart of Him who changes not. And so His people recognise in Him the one satisfying portion of the suffering and sorrowful; as knowing perfectly, understanding completely, entering into the mystery of each trouble, anxiety, and care, more fully than we ourselves can do; so experienced in human affliction, that He can bring with Him, when He comes to our side, the memory of a thousand varied griefs wherewith He was himself exercised; and so filled with tender, soothing, discriminating love, that to those who thus receive Him, sorrow loses its sting and pain its bitterness.—*T. V. Fosbery.*

He is tenderest, not who has sinned, as is sometimes vainly thought—but who has known best the power of sin by overcoming it.—*Westcott.*

Our Heavenly Father is evermore teaching His children, not alone through His Word, His Church, and Sacraments, but by outward circumstances and inward experiences, by days of brightness and seasons of sorrow, bringing out from each, in turn, manifold lessons for such as with lowly, watchful hearts are ever "waiting upon God."

But if this be so, surely He has special teaching for those on whom He, in His infinite wisdom, has seen fit to lay the cross of sorrow, sickness, or suffering. Those who have watched by the bedsides of the sick, as well as those who have had personal experience of pain and weakness, can abundantly testify to the merciful dealings of our compassionate Father at such times. His lessons to his afflicted children are varied, as are the different characters of those whom, by his loving discipline, He is chastening here, that He may fit them for those blessed mansions above, which He meanwhile is preparing for them.

☞ For The Nurses' Looking Glass, Everybody's Opinion, Book World for Women and Nurses, &c., see page ccix. et seq.

### The Muses' Looking Glass.

### "DICK SHERIDAN," COMEDY THEATRE.

It is difficult to realise, as we do from Mr. Buchanan's play, that "The Rivals," that stock success, that hope of amateurs, that play which, like the fabled superior satin gown of our ancestors, can "almost stand alone," should have proved a hopeless and irretrievable failure on the first night at Covent Garden. Yet so it was, and that the disaster was the result of a mean social cabal, formed by young Sheridan's jealous friends, and carried out through the help of a drunken actor, Nat Lee, only shows the improvement in the ethics of things theatrical. A social cabal might be raised nowadays in vain against a play of such really intrinsic merit as "The Rivals."

Mr. Buchanan's version of Dick Sheridan's *début* as a playwright follows fact pretty closely where fact is known. There was, however, a great deal of hazy gossip about his meeting and actual relations with Miss Linley. This improvident, happy-go-lucky youth of genius is first shown us at Bath, among the gay, frivolous folk there. He engaged the affections of the beautiful singer, Elizabeth Linley, and naturally makes many a bitter enemy in so doing, among others a certain Captain Mathews, "a married man"—there fact comes in!—and an old Lord Dazzleton. Both gentlemen have "views" on Miss Linley. In a short while the young lady's mind is effectually poisoned against Dick Sheridan, through the machinations of Lady Miller and Captain Mathews, and the Bath *clique* generally, and she agrees to fly to France under the protection of Mathews. Dick hears of this plan in time to clear his character in her eyes. She elopes with him instead, and they are secretly married there. Here the plot grows hazy. This strong climax in the second act weakens the other two, which are chiefly concerned with Dick's poverty and the production of his play. Miss Linley hardly appears. The duel with Captain Mathew on her account in the fourth act reanimates the spectators' flagging interest and somewhat redeems a singularly ill-constructed play. Mr. Irving comes out strongly. He has his father's strong sense of picturesque pose—of effective attitude. The duel is fought in his threadbare chambers, his faithful servant O'Leary stands in the window seat holding a pair of candelabras; it is a duel to the death, and a sense of grim fatefulness is imparted to it by Mr. Irving, who fights with all the deadly seriousness and *savoir faire* of which he has the tradition.

His acting leaves much to be desired, but all through the play Mr. Irving shows himself a consummate master of pose. First one pose, then another. We see the gallant young playwright swaggering at Bath among the gay folks there, then the scene changes, and he is shown to us in his bare, wretched rooms in London, overwhelmed with debts and waiting anxiously the popular verdict on the child of his witty brain—his last stake, his only hope of being able to openly acknowledge the beautiful Elizabeth Linley as his wife. We see him staggering, at midnight, into Betty Linley's boudoir, with the cold dews of despair breaking out on his forehead to announce its failure, and bid her forget him for ever and consider their marriage null and void. A few days ago we had seen him gay, smiling, and *débonnair*, receiving with the grace of a prince a visit from the great Garrick in the humble garret where the masterpiece had been produced, all unconscious of the cabal which was even then being formed against him. Such a part, so full of contrasting fortunes, of rapid changes of mood, admirably suits Mr. H. B. Irving, who in this piece is given his opportunity. His face is well skilled to express varying emotions, the mobile eyebrows go up and down, his gestures aptly translate, in their capricious alternations, the splendid courage of youth, and the lassitude of despair—in short, he is Henry Irving's son, and it is impossible now and then not to fancy that the veteran actor, grown young again, stands before us, so convincing are the tricks of gesture, the arbitrary inflexions of the voice, and, it must be admitted, the mannerisms which infect the great actor's style. Miss Emery is rapidly making her way to the front of her profession. She speaks her rather high-flown "lines"—could not Mr. Buchanan have given her a touch of sweet, human, commonplace, colloquial nature now and then?—with grace and dignity, and in her lovely trailing silks, and fair hair beaded with loops of pearls, she looks a lady to be fought over and cherished when won. But she can do very little with such a thankless part, except wear her dresses and suffer gracefully. Mr. Cyril Maude, as Lord Dazzleton, creates something of a character part in a crowd of nonentities; but even he is heavily handicapped by eighteenth century trappings and commonplaces of traditional stage affectation. Were all old beaux tiresome and knock-kneed and over-dressed in those days? Mr. Brandon Thomas, as O'Leary, has very little to say, and many jokes of inferior quality to pronounce, which he redeems as far as possible by the irresistibly comic play of feature of which he is master. Let us say a word for Miss Radcliffe in her small part of Lady Shuttleworth. The whole play is a beautiful piece of bric-a-brac, but the breath of life informs it not at all.

### Women's Volunteer Medical Staff Corps.

A meeting was held on February 15th, at 19, Euston Square, N.W., for the purpose of considering the propriety of founding a Women's Volunteer Medical Staff Corps. Mrs. Heatherley was in the chair, and the objects of those interested in the movement were explained by the hon. secretary, Miss Stokes, who said she had no doubt that the corps would be able to render much-needed assistance to the volunteers. As soon as it was formed the members would go through the course prescribed in the "manual" used by the Medical Staff Corps, as well as one of thorough instruction in elementary drill. The question of uniform was also touched upon. It was resolved to call a committee meeting after an interval of three weeks, the members in the meantime doing all in their power to gain recruits and to bring the matter before the notice of the public; on their success in so doing would depend the measures to be decided on at the next meeting.

### Notes and Queries.

### SPECIAL NOTICE.

The contents of the Editor's Letter-box have now reached such unwieldy proportions that it has become necessary to establish a hard and fast rule regarding Answers to Correspondents. In future, all questions requiring replies will continue to be answered in this column without any fee. If an answer is required by letter, a fee of half-a-crown must be enclosed with the note containing the enquiry. We are always pleased to help our numerous correspondents to the fullest extent, and we can trust them to sympathise in the overwhelming amount of writing which makes the new rules a necessity. Every communication must be accompanied by the writer's name and address, otherwise it will receive no attention.

### Queries.

(9) *Dispensing.*—Where can I learn dispensing without relinquishing my present employment?—*T. M.*

(10) *Proba ioners.*—Can anyone give me the names of hospitals where probationers of 20 are taken?—*M. M.*

(11) *Attendant.*—What is the best plan for a young woman to adopt when she wants a post as attendant on an invalid?—*L. K. J.*

### Answers.

(9) *Dispensing (T.M.).*—In "Burdett's Hospital Annual" there is a chapter which gives all particulars. It is headed "Dispensing."

(10) *Probationers (M.M.).*—We do not think there is any general hospital in London which takes such young probationers, although some provincial ones may do so, as well as in Ireland. Children's, Fever, and Special hospitals also take young probationers. Get "How to Become a Nurse" by Honnor Morten, published by Scientific Press, 428, Strand.

(11) *Attendant (L. K. J.).* — Study The Hospital advertisement columns, or advertise yourself.

### Everybody's Opinion.

[Correspondence on all subjects is invited, but we cannot in any way be responsible for the opinions expressed by our correspondents. No communications can be entertained if the name and address of the correspondent is not given, or unless one side of the paper only be written on.]

### CHEAP LABOUR.

"One of the Applicants" writes: Having seen an advertisement for an "assistant matron with some hospital experience," I applied for the post, stating my previous training, age, &c., and inquiring as to the duties appertaining to the vacant post. I naturally imagined them to be somewhat varied and numerous, but the truth so far exceeded my anticipations that I think it may interest readers of The Hospital. I was informed by return of post that "the duties consist in taking entire charge, with the assistance of a doctor when necessary, of the health of the boarders, about forty in number" (it is a novelty for the doctor to be classed as the nurse's assistant!) "waiting upon them and takirg their meals to them when sick. As it often happens that for weeks there are no invalids to attend to, you would be required to fill up your time in assisting the matron in all sorts of light household duties, such as superintending servants, dusting rooms, washing up china and glass, and in the evenings lavatory work with the pupils. On baking days in helping the cookery mistress, and in the laundry ironing pockethandkerchiefs." Therefore, in addition to entire charge of the health of forty girls, a lady has the advantage of doing parlour and housemaid's work; also assist laundry and kitchen maids! "The salary is £16 and laundress, with the option of making this your home for the whole year, &c." But every picture has a bright side, and "the advantages are that you would be treated as a lady, ranking with the governess and matron." Naturally there is an overwhelming attraction in the prospect of being treated as a lady between the intervals of working with and at the pay of the under servants; but I shall resist the temptation, and not feeling that experience and love of hospital work inclines me to take the ambitious position of assistant matron in a ladies' educational establishment.

### COTTAGE NURSES.

"Honorary Secretary Dorset Health Association" writes: The article on "Old-time Nurses" in The Hospital of February 9th exactly expresses my own views. It is in order to attract to our ranks, as cottage nurses, such women as these, the capable, but unfashionable, and no longer good-looking nurse, that the Dorset Health Association has lately opened a county training home for cottage nurses at Blandford. We are convinced that there must be many middle-aged women even in our own county who would welcome the chance of coming there for a few months to prove their capabilities under a first-class hospital-trained nurse, sufficiently enlightened to judge each on her own merits. None are such severe critics as the amateur and ignorant, and we feel that it requires the wise and kindly discrimination of a thoroughly experienced nurse to sift the wheat from the chaff of would-be local nurses. Utterly ignorant of hospital routine, they may have had years of private tuition under country practitioners, as well as that best of all teachers—experience. These two combined have produced nurses whose patience, quick resource, and obedience to orders would put to shame many an impatient, exacting, and self-confident modern nurse. While it is no less true that the typical Mrs. Gamp is not wholly extinct in our rural villages, and most desirable it is that she should be superseded. There are many middle-aged trained nurses with a child dependent on them or who are in some way handicapped, as it were, with the maintenance of some relative, to whom the positions we hope to offer as club or district nurses will ensure a settled home and a small maintenance. There are many other local nurses, not perhaps first-rate, who may apply to us, and our great desire at present is to raise a sufficient fund, either privately or by the help of the County Council, to enable us, after gauging their capacity, to give such training in special subjects as they prove themselves deficient in to fit them for cottage nurses for the smaller clubs. Among younger candidates we hope to come upon gems of "purest ray serene," whom we shall, at the end of their three months' probation, hand on for a regular three years' hospital course, in the full confidence that they will rise to the highest ranks of the profession to which we shall have the honour of introducing them. Anyone wishing to correspond on the subject may apply to the Hon. Secretary Dorset Health Association, Ranston, Blandford, for further information.

### OLD-TIME NURSES.

"The Hon. Secretary Workhouse Infirmary Nursing Association" writes: I have read the article on "Old-Time Nurses" with much interest. It has been a frequent source of regret to me that Boards of Guardians limit the age of candidates for union infirmaries to forty years. In many cases mutual benefit would accrue by the appointment of middle-aged nurses, especially in country unions. I understand that one cause of this limitation of age is the not unnatural fear of the guardians that nurses may become earlier incapacitated for work, and that their pension list may unduly increase, as after a certain number of years, satisfactory service Poor Law officers are entitled to pensions. Many workers feel with me that some change in the system of pensions for nurses working under the Poor Law is highly desirable. If Boards of Guardians were empowered by a Bill to follow the example of hospitals, and pay a part of the premiums of their nurses to the Royal National Pension Fund, much good would be effected; they would be freed from responsibility as regarded the nurses' future, who in their turn could retire from work at a suitable age. Under the present system, a nurse frequently remains in ono union infirmary when her working days are practically over, waiting for a certain date at which she is entitled to a pension. That this condition of things is undesirable in the interests of the patients may be gathered from the case of one nurse whom I knew to be in active (?) service in a country union at the age of seventy years. She remained on in order to secure a pension, which she only lived to enjoy for three months. I am aware that there are many difficulties connected with this question, but as the demand for trained nursing in workhouse infirmaries increases daily, the question must be faced as one of great importance. Under the present conditions guardians are afraid to avail themselves of the services of middle-aged nurses of high character, thorough training, and experience, although they are peculiarly fitted for the care of the aged and infirm, a large proportion of whom are found in country unions. The younger nurse, fresh from her training, and full of a laudable desire for interesting cases and operations, is not unlikely to lose patience with the chronic nature of her work, as well as with the monotony of her surroundings. But to the older nurse, who has seen "active service," the country workhouse infirmary, in which a fair salary is given, should offer a useful field of work, as well as a settled home. Her accumulated patience, tact, age, and experience are precisely the qualities required in dealing with many of the difficult cases met with in infirmaries. However, at present the question of pensions stands in the way, and younger women come and go, frequently staying only for short periods, and giving to guardians a general impression that trained nurses are a restless class. There are undoubtedly other causes that determine this result, but I think that one of the principal reasons would be obviated if the clause relating to age were omitted from Poor Law advertisements for nurses, and one demanding thorough training substituted.

### The Book World for Women and Nurses.

[We invite Correspondence, Criticism, Enquiries, and Notes on Books likely to interest Women and Nurses. Address, Editor, The Hospital (Nurses' Book World), 428, Strand, W.C.]

In a Cornish Village with Old Vogue Folk. By Dolly Pentreath. (T. Fisher Unwin, London.)

There is something very fascinating in the title of this volume of Miss Pentreath's, and quietly as the tale unwinds itself, we are led with interest through the pages, in which the simple records of the old Cornish township are put forth. If there is a clannishness with the people of the north, there is also a kinship recognised amongst the inhabitants of our far western counties. Characteristics, as well as vernacular, are distinct in Cornwall and Devonshire. Both characteristics and vernacular are kept to in the tale, which is told in the words of one of the old vogue folk. He who tells the tale, "one Robert Rowe, for twenty years the parish clerk," makes known the ways and the doings of those he himself knows so well. Squire Pencoose, Madam his wife, his somewhat rough and boorish son, and pretty Fanny Uglow of the farm are living characters, whom we judge, as Robert Rowe would have us judge them, through the quaint and slow moving machinery of his mind. The methods of this mind, the conclusions its owner draws from the events he passes before us, form the *raison d'être* of the book; the plot of the story is not its prominent feature. Adding to the interest of the volume before us are the one or two charming pieces of landscape amongst the illustrations by Percy Craft. The type, paper, and binding are especially attractive.

St. Vesta, or New Worthing. By T. R. E. S. (The Southern Publishing Company (Limited), 62, Fleet Street, E.C., and 130, North Street, Brighton.)

This poem, with sanitation as its motif, might pass without much difficulty for an unpublished Tennysonian idyll—an early one, perhaps, for the full richness of the master's language is not reached, and an occasional line comes near the weakness of the conclusion of "Enoch Arden." But there is no doubt whence came the inspiration of such lines as these :—

> But Vesta held her will against her will,
> Distrusting her own strength and his strong love,
> And would not let him come to say farewell.
>
> Till one day, as the redd'ning sun sank down,
> Because her love was greater than her strength,
> And the pent passion passed beyond control,
> And the slight silver cord was almost loosed,
> And the great fear of separation fell
> Like a dread barrier between world and world—
> Then, in the golden gloaming, Vesta sent,
> And Sanctus, listening for the summons, came.

The story is simple and graceful. Vesta, a princess, has been warned that the confession of her love to the prince to whom she is betrothed will bring about her lover's death, so leaves her home and comes to "St. Vesta's city"—presumably Worthing—in disguise :—

> Some thought she was a princess crossed in love,
> Who gave her life for all instead of one.
> But none knew who she was or whence she came,
> But all knew that she sickened of the plague,
> And to her life's devotion gave her life.
> Another scrap upon the pages tells
> Of Sanctus, one in science deeply versed,
> Who came to help the town in its distress,
> Who drew pure water from the hill-side springs,
> And purged the town from all the cause of death,
> And, broken-hearted, died when Vesta died,
> Leaving great riches to endow the town.

Sanctus is, of course, Vesta's princely lover in disguise; and the souls of the two are permitted, three hundred years after, to revisit the city, cleansed and purified, whose regeneration their self-sacrifice brought about.

The blank verse in which the story is told is, as we have said, largely Tennysonian in rhythm; but that the author has an original gift of song may be gathered from a charming lyric called "St. Vesta's Hymn" :—

> Sleep, sweet sleep, I bring to thee
> By my hand upon thy brow,
> By thy gaze that rests on me,
> By my gaze upon thee now.
>
> Fear not, gently close thine eyes
> While I shade them from the light,
> All the sweetest things we prize
> Steal into our dreams at night.
>
> Feel, I take thy hand in mine,
> Feel, I kiss thy burning brow,
> Feel, I give my life for thine,
> Sleep, sweet sleep, enfolds thee now.

To our readers we commend this poem, if only for the sake of that third speaker in the prologue who says :—

> Among our nurses we could find
> A perfect woman almost anywhere.

To the author we commend the bolder use of a genuine poetic gift and a graceful imagination.

### MAGAZINES OF THE MONTH.

The Englishwoman's Year Book. (F. Kirby, London.)—'The Englishwoman's Year Book" for 1894, is a handy and useful little volume of reference. It is most comprehensive and on the whole very well arranged and indexed—the last, a most important and often unsatisfactory point in these books of references. There are, however, one or two singular omissions in the lists of institutions, especially amongst the nursing institutions for supplying trained nurses for private cases. The most striking of these is the absence of any reference to the Nurses' Co-operation, 8, New Cavendish Street, and the well-known establishment at 123, Bond Street. The omission of the mention of the Nurses' Co-operation is especially unfortunate in the interests of the public. The institution although only established a few years, is already so well-known that it does not resort to much advertizing, consequently for the sake of the public it should be absent from no list of nursing institutions, seeing that the staff already numbering over two hundred nurses, are the most carefully selected in all London, whilst the philanthropic have the satisfaction of knowing that the nurses here obtain the full benefit of their earnings. We would further point out that a list of literature of which a large amount exists especially devoted to hospital and nursing interests would add to the use of the book, which already has attained a popularity, which further completeness will increase.

The Westminster Review for this month is very good reading. An article on "The Coal Question and the Nationalisation of Mines" appeals to most of us who have suffered through the late crisis. Lady Cook writes on "Habits and Customs of Mediæval Times," and though perhaps we find no very new light thrown on this subject, yet it is treated in an interesting and pleasant manner. "Phases of Human Development" is continued by Mona Card, and Mr. G. M. Fraser gives an excellent article on "Shorthand Writing in Foreign Lands." Among Continental people the Germans, he tells us, lead the way in this particular study, the oldest system of shorthand in Germany being an adaptation, published in 1679 at Frankfort, of the English "Tachygraphy" of Thomas Shelton, who flourished as an "author and professor of the said art, in the Poultry, near the church." Mr. Fraser's account of how this art is employed on the Continent is well worth reading.

